# Mode of action and membrane specificity of the antimicrobial peptide snakin-2

**DOI:** 10.7717/peerj.1987

**Published:** 2016-05-10

**Authors:** Vera Herbel, Michael Wink

**Affiliations:** Institute of Pharmacy and Molecular Biotechnology, Ruprecht-Karls-Universität Heidelberg, Heidelberg, Germany

**Keywords:** Snakin-2, Antimicrobial peptide, Pore-forming activity, Non-specific, Bactericide, Fungicide

## Abstract

Antimicrobial peptides (AMPs) are a diverse group of short, cationic peptides which are naturally occurring molecules in the first-line defense of most living organisms. They represent promising candidates for the treatment of pathogenic microorganisms. Snakin-2 (SN2) from tomato (*Solanum lycopersicum*) is stabilized through six intramolecular disulphide bridges; it shows broad-spectrum antimicrobial activity against bacteria and fungi, and it agglomerates single cells prior to killing. In this study, we further characterized SN2 by providing time-kill curves and corresponding growth inhibition analysis of model organisms, such as *E. coli* or *B. subtilis*. SN2 was produced recombinantly in *E. coli* with thioredoxin as fusion protein, which was removed after affinity purification by proteolytic digestion. Furthermore, the target specificity of SN2 was investigated by means of hemolysis and hemagglutination assays; its effect on plant cell membranes of isolated protoplasts was investigated by microscopy. SN2 shows a non-specific pore-forming effect in all tested membranes. We suggest that SN2 could be useful as a preservative agent to protect food, pharmaceuticals, or cosmetics from decomposition by microbes.

## Introduction

Multidrug-resistant microorganisms, which cause thousands of deaths each year, have become more prominent in recent decades, and it is necessary to find and investigate new types of antimicrobial agents to counteract these pathogens. Due to the diversity of potential pathogens, many host defense mechanisms, including antimicrobial peptides (AMPs), have evolved upon infection. These ribosomally synthesized natural antibiotics occur ubiquitously throughout prokaryotes, insects, plants, and animals and can be classified according to various characteristics like biological function, peptide properties as net charge or hydrophobicity, 3D structure, covalent binding patterns, molecular targets, or biological source (Tam et al., 2015). AMPs are a diverse group, but the majority share characteristics like cationic net charge, small molecular weight, and a similar antimicrobial effect, although amino acid sequences and their consequent secondary structures are likewise highly diverse (Jenssen, Hamill & Hancock, 2006). The mode of action of AMPs is based on electrostatic interaction of the negatively charged membrane of bacteria or fungi and the positively charged AMP. Four models have been proposed to explain the formation of pores in biomembranes of bacteria; either a membrane pore is lined with AMPs (“barrel-stave” model,” torroid” model) or the membrane is spanned by an “aggregate” of lipids and peptides (Henzler Wildman, Lee & Ramamoorthy, 2003; Matsuzaki, 1998; Pouny et al., 1992; Zhang, Rozek & Hancock, 2001).

In plants, AMPs represent an evolutionarily conserved component of the innate immune system involved in defense responses. The family of snakin AMPs shows 12 highly conserved cysteines in the C-terminus of the mature peptide that form six disulphide bonds, which are essential for the biological activity (Nahirnak et al., 2012). Snakin-2 (SN2) from tomato (*Solanum lycopersicum*) is a 66-amino-acid-long peptide that is stabilized through six disulphide bonds in the mature protein. We have recently shown that it exhibits antimicrobial activity against bacteria and fungi, destabilizes the target biomembrane, and kills microorganisms. In the trypan blue assay, we could recently show that the mode of action of SN2 is characterized by the formation of pores in the biomembrane of target cells (Herbel, Schäfer & Wink, 2015). In this study, we further characterized the antimicrobial activity of SN2 for a more precise understanding of its mode of action regarding the membrane-specificity, which might be critical for the potential use of snakin as a new antibiotic substance.

## Materials & Methods

### Microbial strains, cell lines, and media

For the microdilution assay, time-kill curves, and growth-inhibition curves, a gram-positive bacterial strain (*Bacillus subtilis*), a gram-negative strain (*Escherichia coli*), and a yeast (*Saccharomyces cerevisiae*) were chosen to characterize the antimicrobial activity of SN2. These organisms are representatives of three major groups of microbes. We used these strains to get an overview of the antimicrobial activity among different microorganisms. Many food-spoiling organisms belong to the group of filamentous fungi, but we excluded this group in this study, because it was characterized in our last study with the representative *Fusarium solani* (Herbel, Schäfer & Wink, 2015). The bacteria were grown at 37 °C in salt-free Luria-Bertani (LB) broth (1% tryptone, 0.5% yeast extract), and the yeast at 28 °C in Sabouraud-Glucose (SAB) broth (2% mycological peptone, 4% D(+)-glucose). 1.5% agar was added to prepare solid media. The pore-forming activity of SN2 in biomembranes was observed using protoplasts derived from a suspension culture of *Nicotiana tabacum* grown in Murashige and Skoog (MS) liquid medium (Murashige & Skoog, 1962) but without kinetin and indolylacetic acid.

### Recombinant expression of SN2 in *E. coli*

The SN2 peptide was expressed in *E. coli* BL21[DE3] as previously described (Herbel, Schäfer & Wink, 2015). Purification was achieved by affinity chromatography using the Äkta start system (GE Healthcare, Solingen, Germany) combined with Bio-Scale Mini Profinity IMAC Cartridges (Bio-Rad, Munich, Germany). The SN2 peptide was expressed as a protein fused to thioredoxin to mask the antimicrobial activity during expression in the bacterial host. After purification of the fusion protein, thioredoxin was removed by TEV protease digestion (Herbel, Schäfer & Wink, 2015).

### Microdilution

The microdilution assay to determine MIC (minimal inhibitory concentration) of recombinant SN2 was used as previously described (Herbel, Schäfer & Wink, 2015). Liquid growth media and microbial suspensions of 1 × 10^6^ cfu/ml (bacteria) or 5 × 10^5^ cfu/ml (yeast) were added to serially diluted SN2 (35-0.01 µM) and incubated for 24 h at 37 °C (bacteria) or 28 °C (yeast). MIC was determined as the lowest concentration without visible growth.

### Time-kill assay

The bactericidal activity of snakin-2 was analyzed using time-kill curves. For these experiments, recombinant SN2 was added in final concentrations of 0.25×, 0.5×, 1×, and 2× of its MIC to bacterial (5 × 10^5^ cfu/ml) or yeast (2.5 × 10^5^ cfu/ml) cultures, and aliquots were taken after 0, 0.5, 1, 3, 6, and 24 h. These were diluted with the respective liquid medium and plated on agar plates. After incubation for 24 h at 37 °C (bacteria) or 28 °C (yeast), the colonies were counted.

### Growth inhibition assay

The inhibitory effect of SN2 on the growth of microorganisms was determined by growth inhibition curves. SN2 was serially diluted (1× MIC – 1/32× MIC) and bacterial (5 × 10^5^ cfu/ml) or yeast (2.5 × 10^5^ cfu/ml) cultures were added. After 0, 3, 6, 7.5, 9, and 24 h, aliquots were taken, and the optical density was measured at 600 nm using a spectrophotometer (WPA Biowave II, Biochrom, Cambridge, UK).

### Salt sensitivity assay

Since AMPs can be salt dependent, this assay was used to determine the inhibition of SN2 activity in the presence of monovalent cations under more physiological conditions. Recombinant SN2 was used in its 1 × MIC, and NaCl or KCl was added to obtain final concentrations of 0, 25, 50, 100, 150, and 300 mM respectively. Bacterial (5 × 10^5^ cfu/ml) or yeast (2.5 × 10^5^ cfu/ml) cultures were added, and the growth was measured as optical density at 600 nm after 9 h. The growth of a control culture (without SN2) was set to 100% growth and consequential as 0% activity. No growth was set to 100% activity of SN2.

### Hemolysis and hemagglutination assay

To estimate a potential risk for humans, if SN2 would be applied as a pharmaceutical drug, the activity of SN2 was tested on mammalian erythrocytes as a proxy for human cells. To study a potential hemolytic and hemagglutinating effect of SN2, 100 µl red blood cells (defibrinated sheep blood, Thermo Scientific, Braunschweig, Germany) were washed three times with 0.4 M mannitol and mixed in a 96-well plate with 100 µl serially diluted SN2 (35-0.01 µM). 0.4 M mannitol and 1% SDS were used respectively as negative (0% hemolysis) and positive controls (100% hemolysis). After incubation at 26 °C for 1 h, the lowest SN2 concentration that caused distinct hemagglutination was visually identified. After centrifugation, the absorbance of the supernatant was determined by measuring the absorption spectrophotometrically at 570 nm. The percentage of hemolysis was calculated as $10{0}^{\ast }({\mathrm{A}}_{SN2}-{\mathrm{A}}_{\text{mannitol}})/({\mathrm{A}}_{SDS}-{\mathrm{A}}_{\text{mannitol}})$ (Ramirez-Carreto et al., 2015).

### Protoplast production and microscopy

Protoplasts were used to analyze the activity against plant membranes to further understand the behavior of SN2 in its host cells and to study the membrane specificity of SN2. Thereby, the potential of SN2 as a pharmaceutical drug can be evaluated. Protoplasts were produced from a suspension cell culture of *Nicotiana tabacum* 4 days after subculturing. 1% cellulase and 0.25% macerozyme were added to 15 ml of the suspension culture and incubated in a petri dish for 2 h at 26 °C, shaking circumspectly. The protoplasts were washed three times with 0.4 M mannitol and used for microscopy (Keyence BZ-9000, KEYENCE Deutschland GmbH, Neu-Isenburg, Germany). SN2 (17 µM final concentration) was added to the protoplasts or undigested tobacco suspension cells, respectively, and photographs were taken with the microscope software (BZII Viewer, KEYENCE Deutschland GmbH, Neu-Isenburg, Germany).

All experiments were repeated three times. In the graphs, the standard deviations from three repetitions are shown as error bars.

## Results

### Bactericidal and fungicidal kinetics of SN2

**Figure 1:**
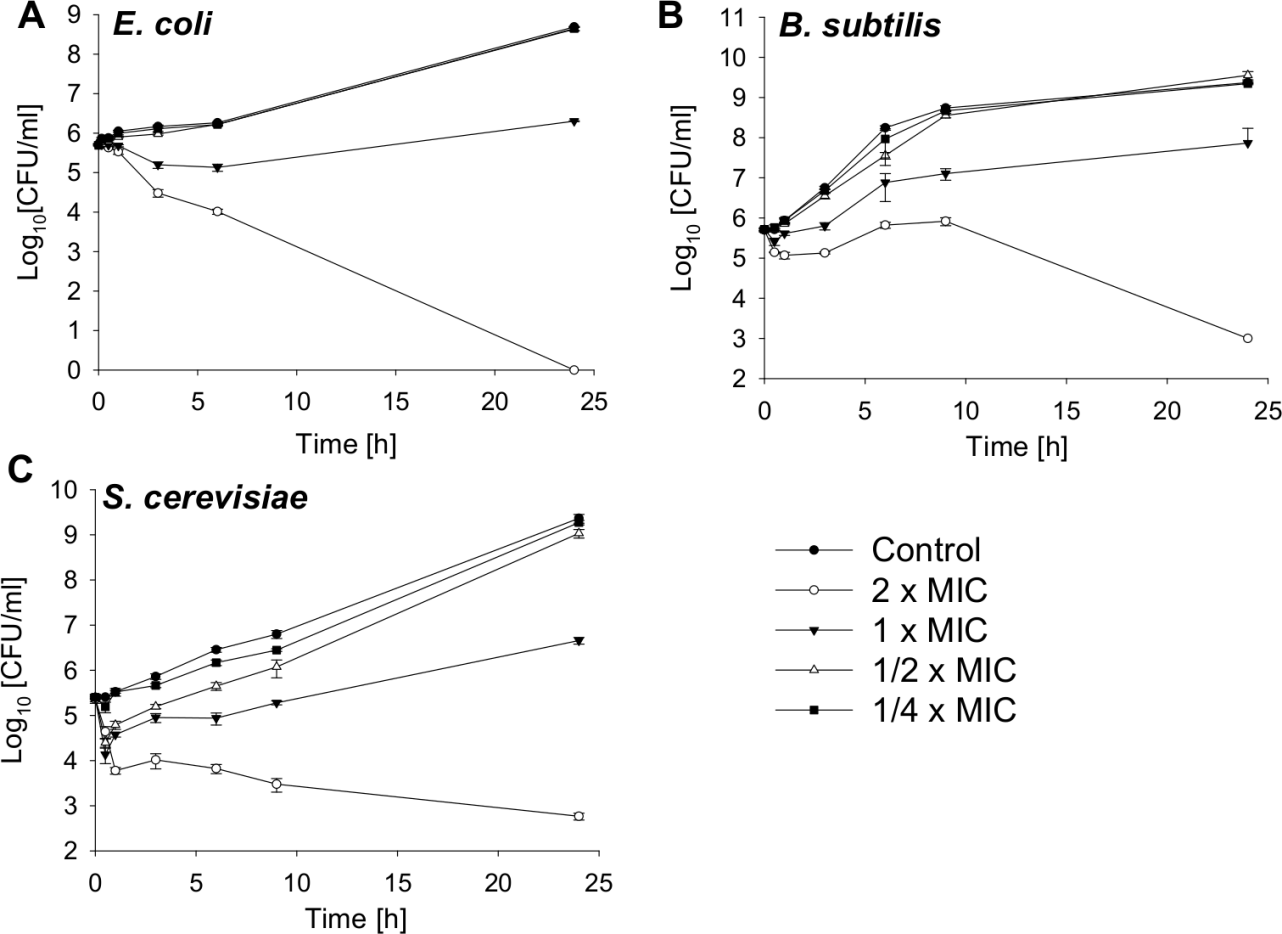
Time-kill curves of SN2 against *E. coli*, *B. subtilis* and *S. cerevisiae*. The cells were treated with varied concentrations of SN2 for different durations and plated on agar plates. Bactericidal or fungicidal activity was determined after 24 h of incubation by counting bacterial or fungal colonies, respectively. Values represent means from three replications ± standard deviation.

Bactericidal activity against a gram-negative and gram-positive bacterial strain and fungicidal effects against a yeast are shown in Fig. 1. The MIC value for *E. coli* is 4.25 µM, for *B. subtilis* 2.12 µM and for *S. cerevisiae* 4.25 µM. The bacterial and yeast cells were treated with SN2 in final concentrations of 2×, 1×, 1/2×, and 1/4× MIC. After 0, 0.5, 1, 3, 6, and 24 h, aliquots were taken and plated on agar plates. After 24 h incubation, the colonies of bacteria and yeast were counted. For *E. coli*, it was previously reported that the 2× MIC is bactericidal (Herbel, Schäfer & Wink, 2015); this was also observed in the kinetics analysis in which, after 24 h, no viable cells could be detected. For the bacterial strains, the 1× MIC is bacteriostatic. Further, there is no significant difference in the number of viable cells among the 1/2× MIC, the 1/4× MIC, and the control. For *S. cerevisiae*, a slight effect between the 1/4× MIC and the control is visible.

### Bacteriostatic and fungistatic activity of SN2

**Figure 2:**
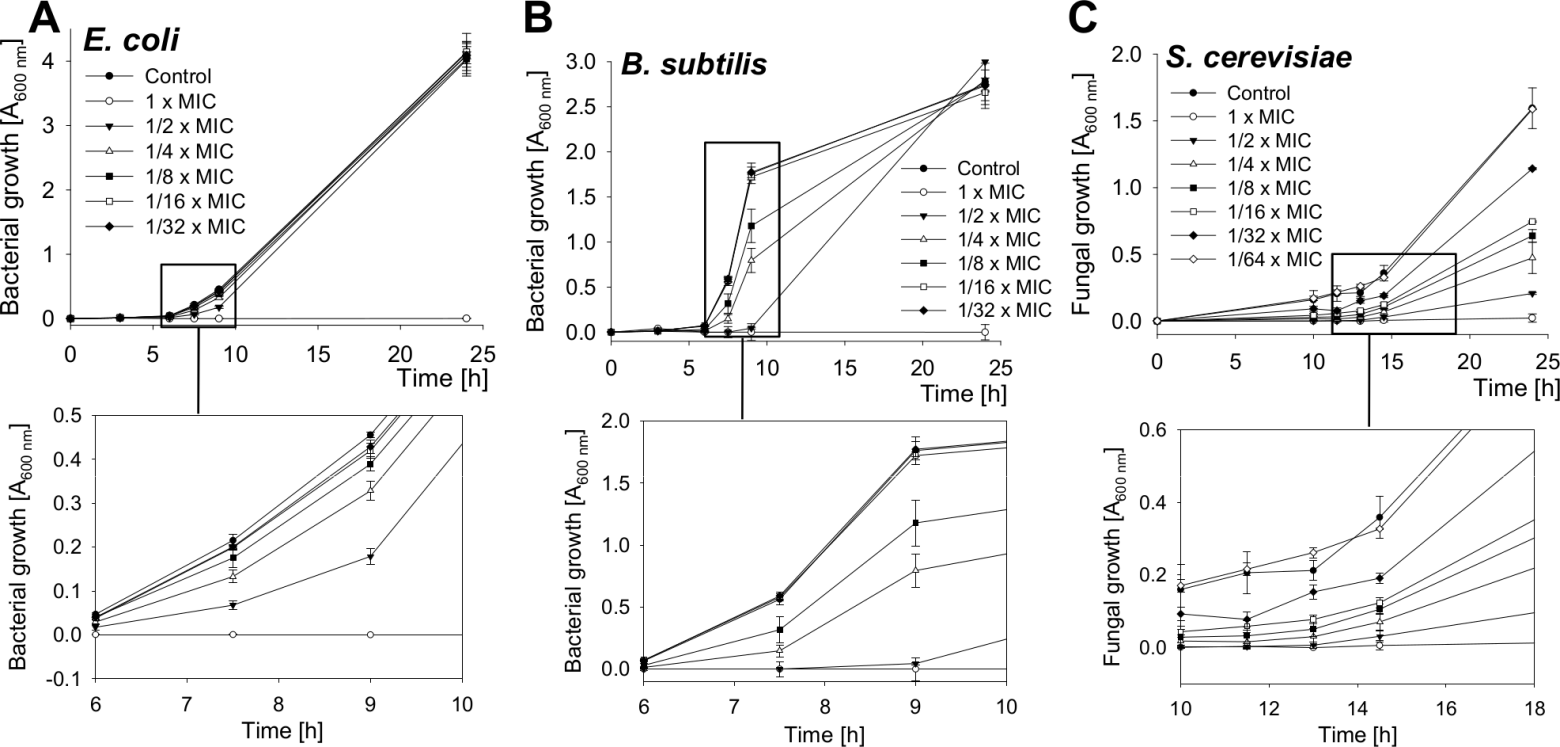
Growth-inhibition curves of *E. coli*, *B. subtilis* and *S. cerevisiae*. Bacterial and yeast cells were treated with several concentrations of SN2, and the growth of the cultures was measured spectrophotometrically. Values represent means from three replications ± standard deviation.

The growth of bacteria and yeast cells in the presence of several SN2 concentrations was analyzed by measuring the optical density at 600 nm after 0, 3, 6, 7.5, 9, and 24 h and is shown in Fig. 2. In the log phase of bacterial growth, a clear inhibition by SN2 is visible. Even the 1/8× MIC, which showed no bactericidal effect, appeared as a concentration that inhibits the growth of both gram-negative and gram-positive bacteria. For the yeast, an even lower concentration of 1/32× MIC affects cell growth compared to the control. The 1× MIC completely inhibits growth of all the tested cells. In comparison to the time-kill curves, it is clear that SN2 early shows growth-inhibiting activity even at low concentrations, but it does not kill cells not below a 16-fold higher (bacteria) or 128-fold higher (yeast) concentration.

### Salt sensitivity of SN2

**Figure 3:**
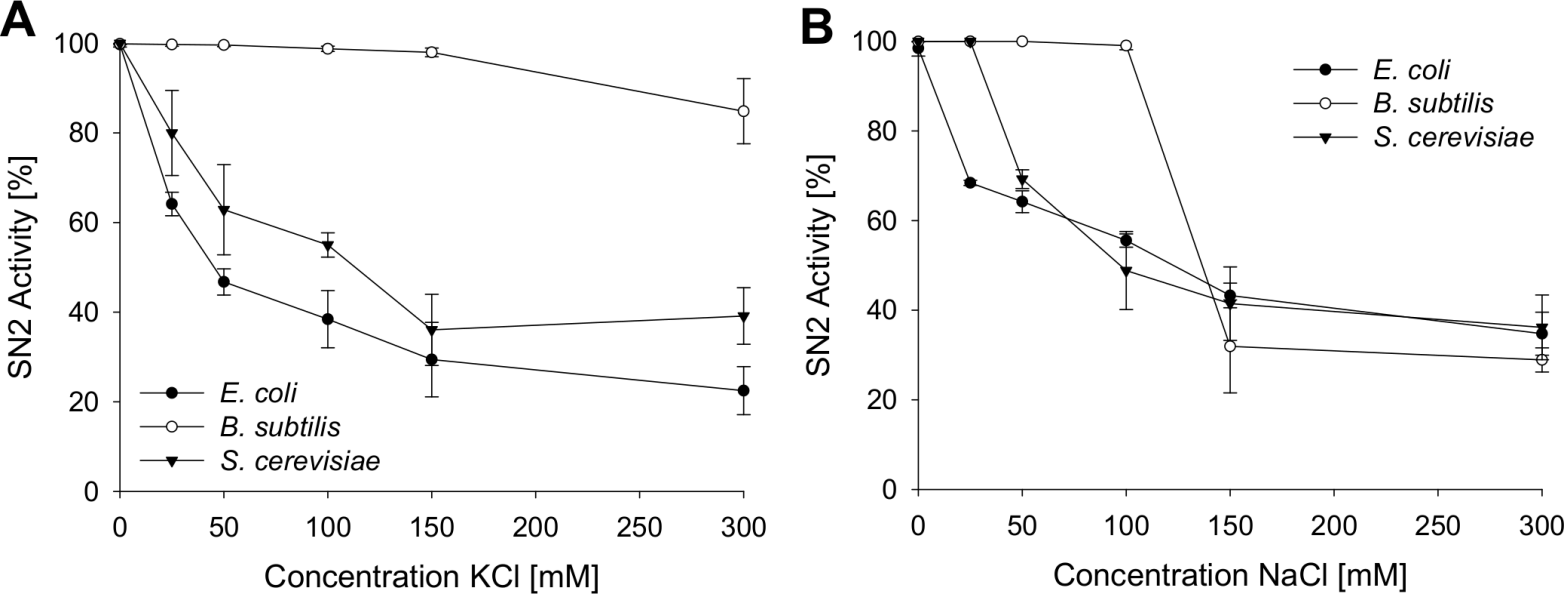
Salt sensitivity of SN2 against *E. coli*, *B. subtilis* and *S. cerevisiae*. The cells were treated with SN2 (concentration of 1× MIC) and varying salt concentrations in the medium. The inhibitory effects of KCl (A) and NaCl (B) on SN2 activity were displayed by measuring the growth of bacterial or yeast cultures, respectively. 100% SN2 activity refers to no bacterial or fungal growth. 0% activity refers to the measured absorption values (600 nm) of a control bacterial or fungal culture without SN2. Values represent means from three replications ± standard deviation.

To analyze the antimicrobial activity of SN2 under physiological conditions, the activity was tested in several salt concentrations. This assay helps to estimate the potential of SN2 as a pharmaceutical drug, because in the human body, SN2 would be exposed to different salt concentrations. Inhibition of SN2 activity by NaCl and KCl is shown in Fig. 3. For all tests, the 1× MIC of the respective strain was used. NaCl or KCl was added in concentrations between 0 and 300 mM to the SN2 solution. Log-phase bacteria or yeast cells were applied, and growth was measured as optical density at 600 nm. 100% activity is referring to a solution without NaCl or KCl. With increasing concentrations of NaCl and KCl, activity of SN2 declined. NaCl and KCl showed similar effects on the reduction of SN2 activity in *E. coli* and *S. cerevisiae*. The SN2 activity against *B. subtilis* was not blocked by KCl concentrations up to 300 mM and NaCl concentrations up to 100 mM.

### Hemolytic and hemagglutinating activity of SN2

**Figure 4:**
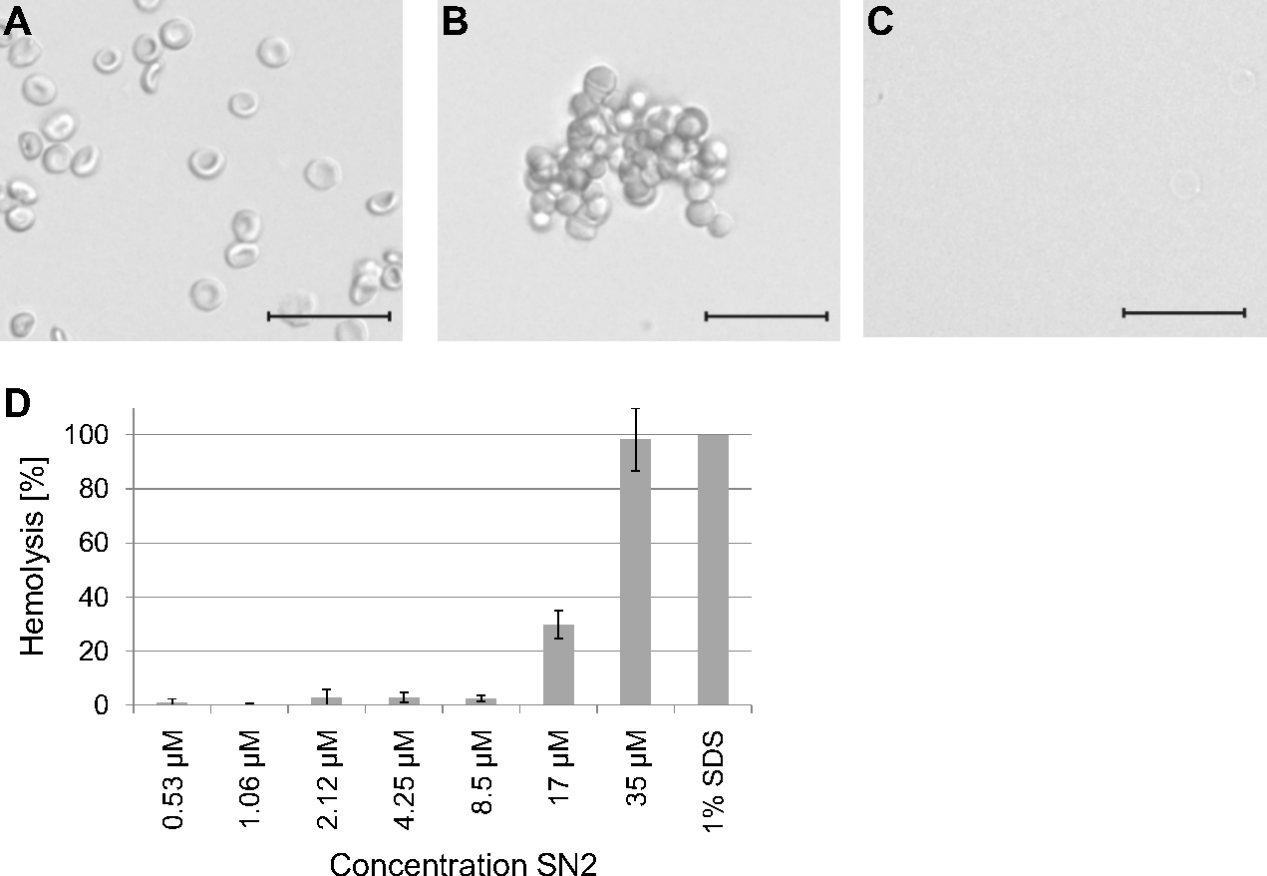
Hemagglutinating and hemolytic activity of SN2. (A) Control sample of sheep erythrocytes in 0.4 M mannitol. (B) Hemagglutinating effect of SN2 after addition of 1 µM SN2 on sheep erythrocytes. (C) Hemolytic activity of 35 µM SN2 on sheep erythrocytes, which are completely lysed. (D) Percentage of hemolysis of sheep erythrocytes after incubation with varying concentrations of SN2 solved in 0.4 M mannitol. The scale bar represents 20 µm.

To estimate a potential risk of SN2 as a pharmaceutical drug, the susceptibility of mammalian erythrocytes was analyzed. The membrane specificity of an AMP is an essential criterion in developing new antibiotic substances. In order to investigate the potential membrane activity of SN2 on mammalian cells, we performed an erythrocyte hemagglutination and hemolysis assay with sheep erythrocytes. SN2 shows an agglutinating effect in the concentration range of 1-17 µM. The agglutinating effect of 1 µM SN2 is shown in Fig. 4B compared with non-treated erythrocytes (Fig. 4A). Hemolysis can be detected at 17 µM SN2 with approximately 30% erythrocytes being lysed, and at 35 µM with 100% hemolysis (Figs. 4C and 4D). No agglutination and hemolysis were observed for the control sample without SN2.

### Effect of SN2 on plant membranes

**Figure 5:**
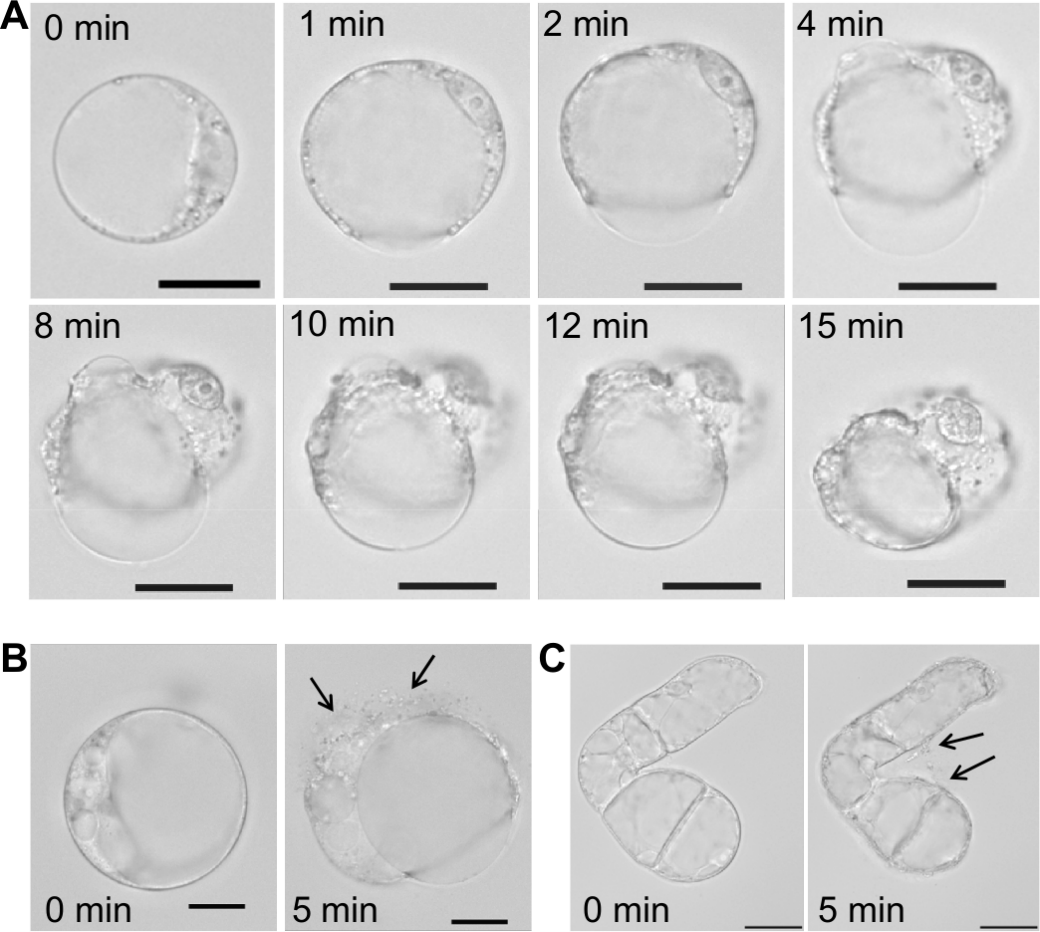
Membrane-active effect of SN2 on plant membranes. (A) Protoplasts, derived from a suspension culture of *Nicotiana tabacum*, shown at different time points after addition of 17 µM SN2 solution. 0 min shows a control protoplast before addition of SN2. (B) Arrows indicating loss of cytoplasmic material from the protoplast 5 min after adding the SN2 solution. (C) Arrows indicating loss of cytoplasmic material from the tobacco suspension cell 5 min after adding the SN2 solution. 0 min shows the cell before treatment with SN2. The scale bar represents 20 µm.

With regard to the unspecific pore-forming effect of SN2, we analyzed the activity of this AMP towards plant cells of *Nicotiana tabacum*, a close relative to tomato, from which the investigated SN2 originates. Protoplasts were used to study the direct interaction of SN2 with plant membranes. The interaction of SN2 with membranes of the pathogenic mold (*F. solani*) has been reported earlier and the membrane perforation could be associated with SN2 molecules through trypan staining of cells with perforated biomembranes. In Fig. 5A, the pictures show the cellular alteration of a protoplast at different time points (1–15 min) after addition of SN2 solution. It was observed that the cell membrane rapidly destabilizes at several points and bursts, so that cytoplasmic material can flow out from the cell. Interestingly, the tonoplast (membrane that surrounds the large vacuole) remained stable for 15 min, and the central vacuole was forced out of the shrinking cell. The loss of cytoplasmic material was documented for both protoplasts and undigested suspension cells (Figs. 5B and 5C). The arrows point out the escaping cytoplasm after 5 min following addition of SN2 solution, indicating that SN2 can diffuse through the plant cell wall, seemingly without problems, and act at the cell membrane.

## Discussion

AMPs become more and more important as a new class of antibiotics, because multi-resistant bacteria present an upcoming threat for humans and domesticated animals. These small peptides are ancient and ubiquitous; they naturally defend most living organisms, which is an essential criterion for a good candidate of novel antibiotic substances (Mansour, Pena & Hancock, 2014). Here, we further characterized SN2, an antimicrobial peptide from the Solanaceae. Snakin peptides defend their host plants against invading pathogens, as shown for StSN1 and StSN2 from potato (Almasia et al., 2008; Berrocal-Lobo et al., 2002; Segura et al., 1999) and for SN2 from tomato (Balaji & Smart, 2012).

Some antimicrobial peptides have been used recently for human or veterinary medical applications like the antimicrobial peptide nisin or polymyxin B (Cao et al., 2007; Hancock & Sahl, 2006; Shin et al., 2015; Yeung, Gellatly & Hancock, 2011). Based on the results from our study, SN2 will not be a useful candidate for medical applications because it acts non-specifically, not only against bacterial and fungal membranes, but also on plant and mammal cell membranes. Even so, it could be applied as a preservative agent to protect food, pharmaceuticals, or cosmetics from decomposition by microbes because, in this case, it is an advantage to act non-specifically against colonizing microorganisms. The salt sensitivity of SN2 is another important aspect because the AMP will become inactive once it enters a human organism containing relatively high salt concentrations. Furthermore, SN2 is a peptide that will presumably be degraded in the human intestinal tract, which ensures patient safety even if it is absorbed by the human body, which makes SN2 unattractive as a candidate for a novel pharmaceutical drug.

Moreover, the results in this study further clarified the mode of action of SN2 regarding the membrane specificity. Through its cationic character, the peptide can diffuse through negatively charged cell walls of bacteria or fungi. Once it reaches the cell membrane, pore-formation commences. Antimicrobial activity decreases after adding salt (NaCl or KCl) to the assay. This could be explained through the saturation of negatively-charged cell wall with cations (Na^+^, K^+^), so that the negative net charge of the cell wall is reduced or reversed. We assume that the cationic AMP is repelled from the cell wall and cannot permeate. The gram-positive *B. subtilis* was more susceptible against SN2, also in high salt concentrations. This effect could be explained by means of different charges of bacterial or fungal cell walls. The gram-positive cell wall contains teichoic acids, which result in a stronger negative charge of the cell wall than in gram-negative bacteria or yeast and could modulate the susceptibility to cationic antibiotics in diverse organisms (Brown, Santa Maria & Walker, 2013; Brown et al., 2012). Therefore, more positively charged ions would be required to cover the negative net charge of the gram-positive cell wall, and this could be the reason that *B. subtilis* stayed more susceptible to the cationic SN2. An inhibitory effect of monovalent and divalent ions has been described before for other AMPs and it was concluded that the mode of action depends on the ionic strength (Lee et al., 2015; Ma et al., 2012). Furthermore, gram-positive bacteria like *Staphylococcus* modulate their teichoic acids by incorporation of D-alanyl residues to neutralize their surface charge to confer resistance against cationic antimicrobial peptides (Saar-Dover et al., 2012; Silhavy, Kahne & Walker, 2010).

The non-specific mode of action of SN2 causes damage even in plant cell membranes, which was shown with tobacco protoplasts in this study. Tobacco plants produce two snakin peptides (SN1 and SN2), which are closely related to those in tomato plants. SN2 rapidly destroyed the cell membrane, but the tonoplast seemed to be less susceptible. This effect has been observed before for the antimicrobial peptide alamethicin. It was suggested that the positive inside membrane potential of tonoplasts (+10 to +40 mV) in comparison to the negative potential of plasma membranes of intact plant cells of −120 mV (Higinbotham, Etherton & Foster, 1967) is causal for the resistance of the tonoplast against the AMP (Matic et al., 2005). Furthermore, it was reported that the tonoplast is multiply folded or shows an sponge-like structure, which leads to a 8-fold larger surface area than that of the plasma membrane (Ryser et al., 1999; Wang et al., 1997). The larger surface of the tonoplast could complicate the destruction of the central vacuole because more SN2 molecules are needed to form pores in all the layers of the tonoplast. This would suggest that SN2 acts like other AMPs as well, in a stoichiometric way, whereby a distinct number of AMP molecules have to conglomerate until a pore can be formed in a biomembrane (Jenssen, Hamill & Hancock, 2006). An alternative explanation of the insusceptibility of the tonoplast against SN2 could be the different lipid composition of plasma membranes and tonoplasts as described for mung beans (Yoshida & Uemura, 1986). In this study, we did not use a label for SN2, as for example GPF could be used, because the activity of SN2 is dramatically reduced if it is fused to another protein, as we found for the expressed fusion protein consisting out of thioredoxin and SN2 (Trx-SN2) (data not shown). Alternatively, a membrane dye could be used, but mostly these dyes should be solved in balanced salt solutions and our results show (Fig. 4), that SN2 would lose its activity in salty solutions. Another option for forthcoming studies is to chemically link SN2 to a fluorescent dye as it was shown before for FITC-labeled lectins or BODIPY-labeled antimicrobial peptides Bac7 and polymyxin B (Benincasa et al., 2009; Strathmann, Wingender & Flemming, 2002).

Through its pore-forming ability, it could be used in combination with other antibiotic substances that target molecules inside the cell as an aid for the other substance to enter pathogenic cells. Thereby the concentration of the antibiotic substances could be reduced, when a synergistic effect would be detectible, as shown before for other pore-forming AMPs (Aleinein, Schäfer & Wink, 2014; Fan, Reichling & Wink, 2013).

## Conclusions

In conclusion, SN2 cannot be applied as a pharmaceutical drug, but it could be a promising candidate for a preservative agent to prolong food, pharmaceutical, or cosmetic storage and to prevent decomposition by microbes by acting non-specifically against colonizing microbes. Due to its non-specific mode of action to form pores in biomembranes of microbes, mammalian and plant cells, and its salt sensitivity, it cannot be used as medication inside the human body, but could be used as antimicrobial agent in topical applications like lotions. Due to the pore-forming activity of SN2, it is a possible candidate for combination studies with antibiotic substances with another cellular target than the biomembrane.

## Supplemental Information

10.7717/peerj.1987/supp-1 Figure S1 Raw Data of Fig. 1 (Time-Kill Curves)

10.7717/peerj.1987/supp-2 Figure S2 Raw Data of Fig. 2 (Growth-Inhibition-Curves)

10.7717/peerj.1987/supp-3 Figure S3 Raw Data of Fig. 3 (Salt-Sensitivity)

10.7717/peerj.1987/supp-4 Figure S4 Raw Data of Fig. 4 (Hemolysis)

## Additional Information and Declarations

### Data Availability

The following information was supplied regarding data availability: The raw data has been supplied as Supplemental Information.
